# Hematological malignancy-associated pyoderma gangrenosum: evaluating the magnitude of the association

**DOI:** 10.3389/fmed.2024.1425454

**Published:** 2024-07-25

**Authors:** Khalaf Kridin, Moria Ankary-Khaner, Mouhammad Kridin, Arnon D. Cohen, Samih Badarny

**Affiliations:** ^1^Unit of Dermatology and Skin Research Laboratory, Galilee Medical Center, Nahariya, Israel; ^2^Azrieli Faculty of Medicine, Bar-Ilan University, Safed, Israel; ^3^Lübeck Institute of Experimental Dermatology, University of Lübeck, Lübeck, Germany; ^4^Department of Ophthalmology, Emek Medical Center, Afula, Israel; ^5^Siaal Research Center for Family Medicine and Primary Care, Faculty of Health Sciences, Ben-Gurion University of the Negev, Beer Sheva, Israel; ^6^Clalit Health Services, Tel-Aviv, Israel; ^7^Department of Neurology, Galilee Medical Center, Nahariya, Israel

**Keywords:** pyoderma Gangrenosum, hematologic malignancies, acute leukemia, multiple myeloma, non-Hodgkin lymphoma, cohort study, case-control study

## Abstract

**Background:**

Hematologic malignancies (HMs) are well-known underlying comorbidities of pyoderma gangrenosum (PG). However, studies quantifying the likelihood of PG after HMs are yet to be performed.

**Objective:**

To investigate the bidirectional association between PG and several HMs, namely acute leukemia, chronic leukemia, Hodgkin lymphoma, non-Hodgkin lymphoma, and multiple myeloma.

**Methods:**

A population-based retrospective cohort study was conducted to study the risk of HMs in patients with PG (*n* = 302) as compared to age-, sex-and ethnicity-matched control subjects (*n* = 1,799). A case–control design was used to estimate the likelihood of PG in individuals with a preexisting history of HMs. Adjusted hazard ratios (HRs) and adjusted odds ratios (ORs) were estimated by Cox regression and logistic regression, respectively.

**Results:**

The prevalence of preexisting HM was higher in patients with PG than in controls (6.7% vs. 0.9%, respectively). The likelihood of having PG was significantly greater among patients with a history of HM (adjusted OR, 7.88; 95% CI, 3.85–16.15; *p* < 0.001), particularly during the first year following the diagnosis. This association was significant for acute leukemia, chronic leukemia, non-Hodgkin lymphoma, and multiple myeloma but not for Hodgkin lymphoma. The incidence rate of HM was 3.3 (95% CI, 1.2–7.4) and 1.6 (95% CI, 0.9–2.6)/1,000 person-years among patients with PG and controls, respectively. Relative to controls, patients with PG were not more likely to develop subsequent HM (adjusted HR, 2.22; 95%CI, 0.77–6.45; *p* = 0.142). Compared to other patients with PG, those with HM-associated PG experienced an increased all-cause mortality rate (adjusted HR, 2.19; 95%CI, 1.09–4.40; *p* = 0.028).

**Conclusion:**

HM, particularly acute leukemia and multiple myeloma, are associated with an elevated likelihood of provoking PG.

## Introduction

Pyoderma gangrenosum (PG) is a rare neutrophilic dermatosis that presents with rapidly developing, painful skin ulcers hallmarked by undermined borders and peripheral erythema (1). Seventy-eight percent of PG ulcers occur on the lower leg, with the most common location being the pretibial area (2). PG associates with multiple comorbidities and has been found to be associated with increased mortality (2, 3). Such comorbidities include inflammatory bowel disease (IBD), hematological disorders, autoimmune and autoinflammatory diseases (4–6).

Hematological malignancy (HM) comprises a collection of heterogeneous conditions, all originating from cells of the bone marrow and the lymphatic system. There are three major groups: leukemia, lymphoma, and plasma cell neoplasms. In Western countries, the overall incidence of hematological malignancies appears to be rising (7). In recent years, medical society witnessed a substantial advancement in therapeutic arsenal for these conditions (8).

In a recent meta-analysis, HMs were ranked as the third most common underlying conditions preceding the onset of PG (6). A systemic review of 279 publications revealed that myelodysplastic syndrome was the most commonly reported HM in association with PG, followed by monoclonal gammopathy of undetermined significance and acute myeloid leukemia (9). Despite this piece of knowledge, controlled observational studies are still missing in order to quantify the likelihood of PG after HM. In the present study, we aimed to investigate the bidirectional associations of PG with HM and evaluate whether patients with PG and comorbid HM have distinct prognostic outcomes.

## Methods

### Study design and database

The objective of this study was to explore the two-way relationship between PG and HM. To assess the risk of developing HM following a diagnosis of PG, we employed a retrospective cohort study design, in which patients with PG were followed over time to estimate the incidence of HM. Additionally, to investigate the likelihood of developing PG in individuals with a prior history of HM, we utilized a case-control study design to examine the prevalence of preexisting HM (as the exposure) among patients who later developed PG (as the outcome) (10). The rare disease assumption’ theorizes that estimates derived from case–control studies focused on rare diseases (defined as those with a prevalence rate of less than 10%) tend to approximate those produced by cohort studies (10). Therefore, the case-control study design used in this research enables us to evaluate the risk of developing PG in individuals with a prior history of HM.

This study made use of the computerized database provided by Clalit Health Services (CHS). As of October 2018, CHS served a substantial population of 4,927,000 enrollees, establishing itself as the largest health maintenance organization in Israel. Further information about the dataset’s specific characteristics can be found in our previous publications (11). The current study received approval from the Institutional Review Board (IRB) of Ben-Gurion University, adhering to the principles set forth in the Declaration of Helsinki (approval code: 0212-17-COM in 2017).

### Study population and main variables

A thorough review of the CHS dataset was conducted to identify all individuals, including pediatric and adult patients, who were diagnosed with PG between 2000 and 2018. Each case underwent a detailed evaluation, and only those that met at least one of the following eligibility criteria were ultimately included: (i) a documented PG diagnosis recorded at least twice by a board-certified dermatologist and/or (ii) the presence of a PG diagnosis in the discharge letters of patients who had received hospital care in a dermatological ward.

The HM variable was defined as the occurrence of one of the following 5 diseases: acute leukemia, chronic leukemia, Hodgkin lymphoma, non-Hodgkin lymphoma, and multiple myeloma. The diagnosis of each one of the HMs was based on its documentation in the chronic disease registry of CHS. To provide further clarification, the diagnosis of HM relied on documentation by board-certified hematologists, the prescription of drugs related to HM, and supportive laboratory findings. This diagnosis was subsequently verified by the primary healthcare provider overseeing the patient’s care. In instances where individuals had more than one type of HM, the date of the initial disease diagnosis was utilized to calculate the index date. Our analyses were run on the pooled HM variable and on each one of its 5 components separately.

The control group encompassed up to 5 controls per every patient, matched randomly by age, sex, and ethnicity. The date of diagnosis was considered the index date for both the cases and the corresponding matched control patients. Prior to their inclusion in the study, controls were verified to be alive and to have contributed longitudinal data to the CHS dataset.

Outcome measures were controlled for the Charlson comorbidity index, a well-established epidemiological tool utilized to evaluate the severity and significance of concurrent medical conditions. This index is frequently used in epidemiological studies and has displayed strong reliability in forecasting mortality (12). To eliminate bias, we used a modified version of the score following the exclusion of the malignancy component of the scoring system. A sensitivity analysis was performed to ensure that the observed association was not overinflated by potential ascertainment bias. This analysis entailed revisiting the calculations while omitting data from the two-year period preceding the index date (the date of PG diagnosis and recruitment of controls). As a result, all individuals who were diagnosed with HM during this excluded period were not included in the calculations.

### Statistical analysis

Baseline characteristics were described by means and standard deviations (SD)s for continuous variables, while categorical values were signified by percentages. To compare sociodemographic and clinical factors between cases and controls, we employed the Chi-square test for categorical variables and the *t*-test for continuous variables.

In the cohort study design, we computed the incidence rates of HM for both PG patients and controls, and these rates were expressed as the number of events per 1,000 person-years. To evaluate the risk of developing new cases of HM, we employed the Cox regression model to calculate hazard ratios (HRs). In the case-control study design, we utilized logistic regression to determine odds ratios (ORs) and their corresponding 95% confidence intervals (CIs). This association was computed based on individuals who developed PG after the diagnosis of each specific HM, taking into account the temporal relationship between exposure and outcome in case–control studies. Statistical significance was defined as two-tailed *p*-values below 0.05. All statistical analyses were performed using SPSS software, version 25 (SPSS, Armonk, NY: IBM Corp).

## Results

### Characteristics of the study population

The present study involved 302 individuals with PG and 1,497 matched control subjects. The mean (SD) age at the diagnosis of patients and enrollment of control subjects was 54.0 (20.8) years. Among patients with PG, 175 (57.9%) were females, and 255 (84.4%) had a Jewish ethnic background. The two groups were statistically similar in terms of gender and ethnic composition, as well as in terms of average body mass index (BMI) and the prevalence of smoking. The characteristics of the study population are detailed in Table 1.

**Table 1:** Descriptive characteristics of the study population.

Characteristic	Patients with pyoderma gangrenosum (*N* = 302)	Controls (*N* = 1,497)	*p* value
Age, years			
Mean ± SD	54.0 ± 20.8	54.0 ± 20.8	1.000
Median (range)	55.8 (0.2–95.1)	55.9 (0.2–95.6)	
Pediatric patients (<18 years; *N* [%])	13 (4.3)	65 (4.3)	1.000
Male sex, *N* (%)	127 (42.1)	629 (42.0)	0.974
Ethnicity, *N* (%)			
Jews	255 (84.4)	1,264 (84.4)	1.000
Arabs	47 (15.6)	233 (15.6)	
BMI, mg/kg^2^			
Mean ± SD	28.0 ± 6.3	27.8 ± 6.2	0.614
Smoking, *N* (%)	115 (38.1)	521 (34.8)	0.274
Charlson comorbidity score			
Mean score ± SD	2.3 ± 2.7	1.3 ± 1.8	**<0.001**
None (0)	111 (36.8)	777 (51.9)	**<0.001**
Moderate (1–2)	78 (25.8)	432 (28.9)	0.276
Severe (≥3)	113 (37.4)	288 (19.2)	**<0.001**

PG, pyoderma gangrenosum; *N*, number; SD, standard deviation; BMI, body mass index.

### The odds of PG following HM

A case-control study was conducted to clarify whether a history of HM places patients at an increased likelihood of developing PG (Table 2). The prevalence of preexisting HM was greater in patients with PG than in control (6.7% vs. 0.9%, respectively; *p* < 0.001). Thus, the likelihood of developing PG after being diagnosed with HM was increased more than sevenfold (OR, 7.58; 95% CI, 3.78–15.19). In an age-, sex-, and ethnicity-stratified analysis, the odds of PG after HM were prominently heightened among older individuals, males, and Jews (Table 2). In a time-stratified analysis, the highest odds of PG occurred during the first year following the diagnosis of HM and decreased gradually thereafter (Table 2).

**Table 2:** The odds of pyoderma gangrenosum among patients with a preexisting history of hematologic malignancy, stratified by age, sex, and ethnicity (case-control study design).

Subgroup	HM in patients with PG; *n* (%)*	HM in controls; *n* (%)*	OR (95%CI)	Univariate *p* value
**All**	20 (6.7%)	14 (0.9%)	7.58 (3.78–15.19)	**<0.001**
**Age, years**				
<54	6 (4.3%)	1 (0.1%)	31.21 (3.73–261.33)	**<0.001**
≥54	14 (8.9%)	13 (1.7%)	5.82 (2.68–12.65)	**<0.001**
**Sex**				
Male	11 (8.9%)	7 (1.1%)	8.61 (3.27–22.68)	**<0.001**
Female	9 (5.2%)	7 (0.8%)	6.67 (2.45–18.17)	**<0.001**
**Ethnicity**				
Jews	18 (7.2%)	12 (1.0%)	8.02 (3.81–16.88)	**<0.001**
Arabs	2 (4.3%)	2 (0.9%)	5.09 (0.70–37.07)	0.075
**Time since diagnosis**				
1st year	5 (1.7%)	1 (0.1%)	25.39 (2.96–218.15)	**<0.001**
1–5 years	4 (1.3%)	1 (0.1%)	20.25 (2.26–181.79)	**<0.001**
5–10 years	6 (2.0%)	6 (0.4%)	5.08 (1.63–15.86)	**0.002**
>10 years	5 (1.7%)	6 (0.4%)	4.22 (1.28–13.91)	**0.010**

PG, pyoderma gangrenosum; HM, hematologic malignancies; OR, odds ratio; *n*, number; CI, confidence interval. *The prevalence of HM in cases when HM preceded PG (in cases) or preceded enrollment (in controls). **Bold:** significant value.

To assess whether there is an independent association between a preexisting history of HM and the development of subsequent PG, we performed a multivariate logistic regression analysis, which controlled for demographic factors and comorbidities. Consistent with the findings from the univariate analysis, a previous diagnosis of HM was found to be independently associated with significantly increased odds of PG (adjusted OR, 7.88; 95% CI, 3.85–16.15; *p* < 0.001).

We then carried out a sensitivity analysis after excluding patients diagnosed with HM up to 2 years before PG. The elevated likelihood of PG after HM maintained its statistical significance both in the univariate (OR, 5.72; 95% CI, 2.66–12.29; *p* < 0.001) and multivariate (adjusted OR, 5.84; 95% CI, 2.65–12.86; *p* < 0.001) analyses.

### The risk of HM among patients with PG

A retrospective cohort study followed patients with PG and controls longitudinally and estimated the incidence of new-onset HM (Table 3). Overall, 5 incident cases of HM occurred among patients with PG and 13 cases among controls. Taken together, the incidence rate of HM was 3.31 (95% CI, 1.21–7.35) and 1.55 (95% CI, 0.86–2.58)/1,000 PY among patients with PG and controls, respectively.

**Table 3:** The risk of hematologic malignancies among patients with pyoderma gangrenosum (retrospective cohort study design).

	PG	Controls
Follow-up time, PY	1,508.9	8,386.9
Median follow-up time, years (range)	4.9 (0.0–17.8)	5.4 (0.1–17.8)
Number of HM	5	13
Incidence rate / 1,000 PY (95% CI)	3.31 (1.21–7.35)	1.55 (0.86–2.58)
Crude HR (95% CI) [*p* value]	2.13 (0.76–5.96) [0.152]	Reference
Male-specific crude HR (95% CI) [*p* value]	**5.77 (1.16–28.62) [0.032]**	Reference
Female-specific crude HR (95% CI) [*p* value]	1.09 (0.24–4.97) [0.913]	Reference
Age-and sex-adjusted HR (95% CI) [*p* value]	2.29 (0.82–6.44) [0.116]	Reference
Fully adjusted HR (95% CI)^a^ [*p* value]	2.22 (0.77–6.45) [0.142]	Reference

a Multivariate Cox regression model adjusting for age, sex, ethnicity, and comorbidities.

PG, pyoderma gangrenosum; *N*, number; PY, person-years; HR, hazard ratio; CI, confidence interval. **Bold:** significant value.

The unadjusted risk of developing HM was comparable between cases and controls (HR, 2.13; 95% CI, 0.76–5.96; *p* = 0.152). The aforementioned risk was of statistical significance only among males (HR, 5.77; 95% CI, 1.16–28.62; *p* = 0.032), but lost its significance in the multivariate analysis that adjusted for putative confounders (fully-adjusted HR, 2.22; 95% CI, 0.77–6.45; *p* = 0.142; Table 3).

### Granular analysis of the association between pyoderma gangrenosum and different HMs

Table 4 demonstrates the association between PG and 5 different types of HMs. An independently significant association was found between PG and acute leukemia (adjusted OR, 11.07; 95% CI, 3.28–37.30; *p* < 0.001), chronic leukemia (adjusted OR, 5.23; 95% CI, 1.93–14.67; *p* = 0.001), non-Hodgkin lymphoma (adjusted OR, 4.50; 95% CI, 1.98–10.24; *p* < 0.001), and multiple myeloma (adjusted OR, 6.30; 95% CI, 1.63–24.32; *p* = 0.008). Interestingly, PG was not associated with Hodgkin lymphoma (adjusted OR, 1.48; 95% CI, 0.26–8.40; *p* = 0.661).

**Table 4:** The association between pyoderma gangrenosum and different hematologic malignancies.

	*N* in PG (%)*	*N* in controls (%)**	OR (95%CI)	Univariate *p* value	Adjusted OR (95%CI)^a^	Multivariate *p* value	Goodness of fit test (Hosmer-Lemeshow Test)
Acute leukemia	9 (3.0%)	4 (0.3%)	11.47 (3.51–37.48)	**<0.001**	11.07 (3.28–37.30)	**<0.001**	0.027
Chronic leukemia	8 (2.6%)	8 (0.5%)	5.07 (1.89–13.60)	**<0.001**	5.32 (1.93–14.67)	**0.001**	0.792
Hodgkin lymphoma	2 (0.7%)	5 (0.3%)	1.99 (0.38–10.30)	0.403	1.48 (0.26–8.40)	0.661	0.343
Non-Hodgkin lymphoma	12 (4.0%)	13 (0.9%)	4.72 (2.13–10.46)	**<0.001**	4.50 (1.98–10.24)	**<0.001**	0.358
Multiple myeloma	5 (1.7%)	4 (0.3%)	6.28 (1.68–23.54)	**0.002**	6.30 (1.63–24.32)	**0.008**	0.591

*n*, number; HM, hematologic malignancies; OR, odds ratio; CI, confidence interval; NA, not applicable.

a Multivariate logistic regression model adjusting for age, sex, ethnicity, and comorbidities.

*11 PG patients developed more than a single HM. **7 controls developed more than a single HM.

### Prognosis of patients with HM-related PG relative to other patients with PG

**Figure 1:**
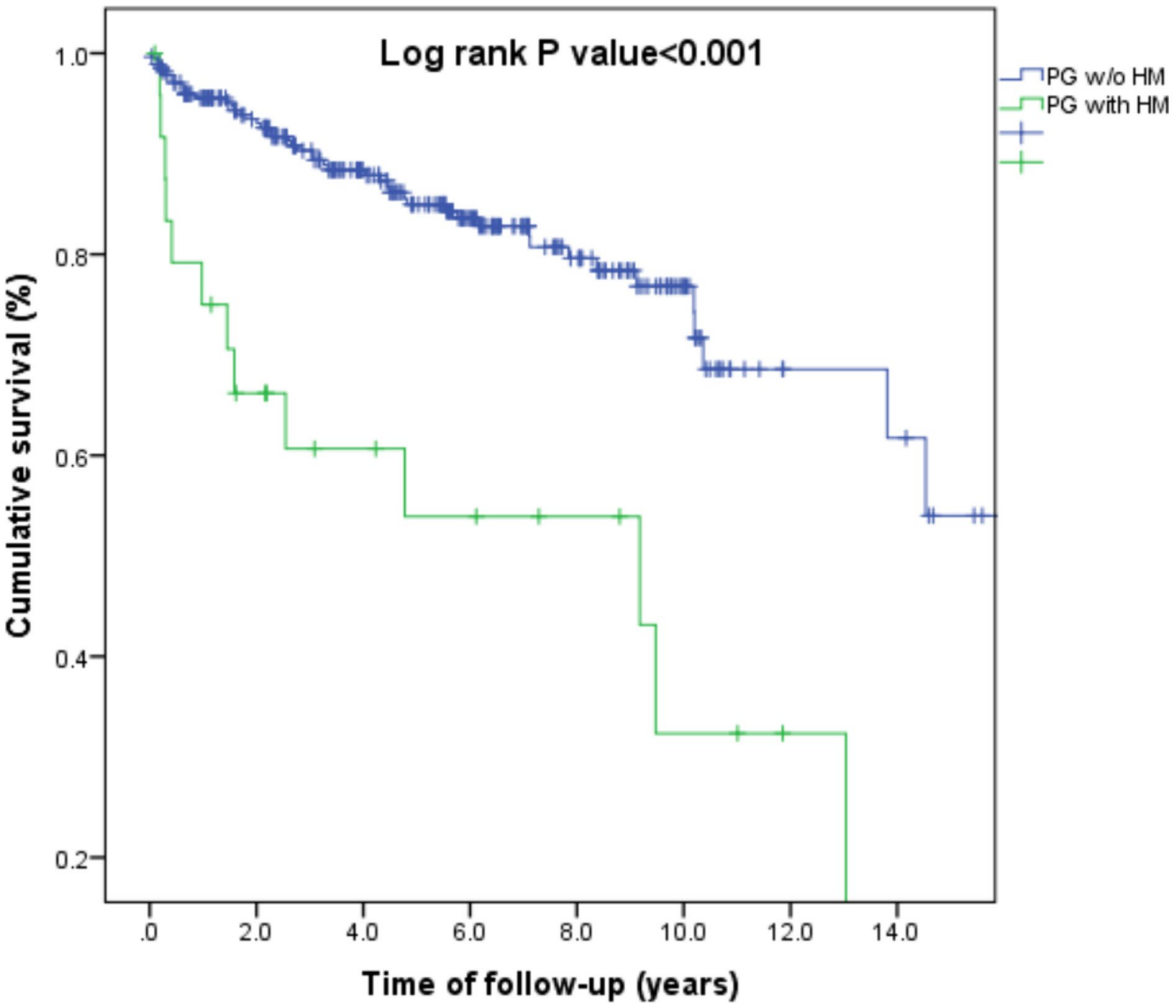
Kaplan-Meier curve demonstrating differences in survival rates between patients with pyoderma gangrenosum and hematologic malignancies as compared to those with pyoderma gangrenosum without hematologic malignancies.

We then addressed the all-cause mortality rate of patients with HM-associated PG (*n* = 25) as compared to the remaining patients with PG (*n* = 277). Patients with a dual diagnosis of PG and HM experienced an elevated risk of all-cause mortality, both in univariate (HR, 3.93; 95% CI, 2.11–7.29; *p* < 0.001) and multivariate (adjusted HR, 2.19; 95% CI, 1.09–4.40; *p* = 0.028) analysis (Figure 1).

## Discussion

The current report marks the inaugural population-based investigation into the two-way relationship between PG and HM. Our results indicate that a prior history of HM significantly increases the likelihood of developing PG by nearly eight times. The highest odds of PG occurred during the first year following the diagnosis of HM and decreased gradually thereafter. In a granular analysis, this association appeared strongest for acute leukemia, multiple myeloma, chronic leukemia, and non-Hodgkin lymphoma, though not for Hodgkin lymphoma. On the other hand, the risk of subsequent HM was not statistically elevated following the diagnosis of PG. Patients with HM-associated PG experienced a 2.2-fold elevated risk of all-cause mortality relative to the remaining patients with PG.

The rare nature of PG interferes with investigating the comorbidities of the condition. As a result, the relationship between PG and HM remains sparsely elucidated. Over the years, the accumulation of case reports and case series regarding the coexistence of the two conditions implies a plausible link between them (13, 14). However, this association was not quantified by a well-designed population-based study. Utilizing data from case reports, case series, and retrospective studies, a recent systemic review detected 340 PG cases associated with HMs (9). Myelodysplastic syndrome (MDS) was the most commonly reported hematologic malignancy associated with PG, followed by monoclonal gammopathy of undetermined significance (MGUS) and acute myeloid leukemia (AML) (9). A systematic review and meta-analysis, pooling data from 21 relevant studies involving 2,611 PG patients, revealed that HMs represented the third most common underlying comorbidity in PG, occurring in 8.9% of eligible patients (6).

Patients with HM experience a large spectrum of skin manifestations, including neutrophilic dermatosis, such as PG. Neutrophilic dermatoses are typified by neutrophil-rich infiltrate on histopathology (5, 15). Recent investigations show myeloid malignancies are the HMs most frequently associated with neutrophilic dermatosis. The connection between these two conditions might be interpreted by the growing evidence suggesting that neutrophils infiltrating the skin may share a clonal relationship with the myeloid neoplastic cells and could have originated from them (15, 16).

The pathophysiology of PG mostly involves innate immunity, though recent evidence suggests adaptive immunity exert a pathogenic role in this disease (15, 17). The initial T cell-dominant infiltrate of PG is mostly associated with T-helper (Th)-1 and Th-17 cytokines, whereas a prominent downregulation of the Th2-promoting transcription factor GATA3 was observed (18, 19). Another putative mechanistic interpretation linking PG with HMs lies in the fact that both innate and adaptive immunity play a pathogenic role in the development of PG. We suggest that increased Th-17 activity, found both in PG and lymphoid malignancies and acute leukemia, might contribute to the observed association (15). The increased risk of all-cause mortality among patients with HM-associated PG relative to other patients with PG is conceivable in light of the high mortality burden imposed by HM by itself (20). HMs entail a higher mortality burden as compared to other underlying comorbidities of PG, like inflammatory bowel disease and rheumatoid arthritis (21).

The case–control design enables the identification of the temporal relationship between diagnoses and allows the estimation of a causal relationship between the two conditions of interest (22). The population-based design and the recruitment of cases managed both in inpatient and outpatient settings minimize the likelihood of selection bias that may arise in other observational studies. One of the main drawbacks interfering with the current study is the lack of data regarding the clinical characteristics and severity indices of the two diseases. Although the formal disease criteria were not directly required to recruit patients, the inclusion criteria were strict and valid; based on documentation by a certified dermatologist or dermatological wards for PG and on the chronic disease registry of CHS for HM. The latter was proven highly reliable in previous studies (23). Furthermore, while the associations between pyoderma gangrenosum and most of the different hematologic malignancies were found to be significant, the small number of patients included in this analysis limited our power to draw definite conclusions. Thus, more investigations are required to understand these relationships fully.

In conclusion, the current population-based study demonstrates that a history of HM confers an eightfold elevated odds of PG. Patients with HM-related PG are at an elevated risk of mortality relative to other patients with PG. Prospective studies are necessary to reproduce our findings in other study populations. Physicians managing patients with HM should be aware of these increased odds of PG, particularly during the first year after the onset of HM. Patients with HM should be advised to avoid additional triggering factors of PG, namely pathergy phenomenon derived from piercing trauma, or unnecessary surgical procedures.

## Data availability statement

The raw data supporting the conclusions of this article will be made available by the authors, without undue reservation.

## Author contributions

KK: Conceptualization, Data curation, Formal analysis, Funding acquisition, Investigation, Methodology, Project administration, Resources, Software, Supervision, Validation, Visualization, Writing – original draft, Writing – review & editing. MA-K: Data curation, Formal analysis, Writing – original draft. MK: Conceptualization, Visualization, Writing – original draft. AC: Investigation, Resources, Writing – original draft, Writing – review & editing. SB: Methodology, Project administration, Visualization, Writing – review & editing.

## References

[1] Maverakis E Marzano AV Le ST Callen JP Brüggen MC Guenova E. Pyoderma gangrenosum. Nat Rev Dis Primers. (2020) 6:1–19. doi: 10.1038/s41572-020-0213-x 33033263

[2] Barbe M Batra A Golding S Hammond O Higgins JC O’Connor A. Pyoderma Gangrenosum: a literature review. Clin Podiatr Med Surg. (2021) 38:577–88. doi: 10.1016/J.CPM.2021.06.002 34538436

[3] Ben Abdallah H Fogh K Vestergaard C Bech R. Pyoderma Gangrenosum and interleukin inhibitors: a semi-systematic review. Dermatology. (2022) 238:785–92. doi: 10.1159/000519320, PMID: 34710873

[4] Binus AM Qureshi AA Li VW Winterfield LS. Pyoderma gangrenosum: a retrospective review of patient characteristics, comorbidities and therapy in 103 patients. Br J Dermatol. (2011) 165:1244–50. doi: 10.1111/J.1365-2133.2011.10565.X, PMID: 21824126

[5] Delaleu J Lepelletier C Calugareanu A de Masson A Charvet E Petit A. Neutrophilic dermatoses. Rev Med Interne. (2022) 43:727–38. doi: 10.1016/J.REVMED.2022.06.007 35870984

[6] Kridin K Cohen AD Amber KT. Underlying systemic diseases in pyoderma Gangrenosum: a systematic review and meta-analysis. Am J Clin Dermatol. (2018) 19:479–87. doi: 10.1007/s40257-018-0356-7, PMID: 29721816

[7] Rodriguez-Abreu D Bordoni A Zucca E. Epidemiology of hematological malignancies. Ann Oncol. (2007) 18:i3–8. doi: 10.1093/annonc/mdl443 17311819

[8] Shimada A. Hematological malignancies and molecular targeting therapy. Eur J Pharmacol. (2019) 862:172641. doi: 10.1016/J.EJPHAR.2019.172641 31493406

[9] Montagnon CM Fracica EA Patel AA Camilleri MJ Murad MH Dingli D. Pyoderma gangrenosum in hematologic malignancies: a systematic review. J Am Acad Dermatol. (2020) 82:1346–59. doi: 10.1016/J.JAAD.2019.09.032, PMID: 31560977

[10] Greenland S Thomas DC. On the need for the rare disease assumption in case-control studies. Am J Epidemiol. (1982) 116:547–53. doi: 10.1093/oxfordjournals.aje.a113439, PMID: 7124721

[11] Kridin K Zelber-Sagi S Comaneshter D Cohen AD. Coexistent solid malignancies in pemphigus. JAMA Dermatol. (2018) 154:435–40. doi: 10.1001/jamadermatol.2017.6334, PMID: 29453868 PMC5876857

[12] Charlson ME Pompei P Ales KL Mac Kenzie CR. A new method of classifying prognostic comorbidity in longitudinal studies: development and validation. J Chronic Dis. (1987) 40:373–83. doi: 10.1016/0021-9681(87)90171-8, PMID: 3558716

[13] Wayte JA Rogers S Powell F. Pyoderma gangrenosum, erythema elevatum diutinum and IgA monoclonal gammopathy. Australas J Dermatol. (1995) 36:21–3. doi: 10.1111/J.1440-0960.1995.TB00919.X, PMID: 7763217

[14] Fox LP Geyer AS Husain S Grossman ME. Bullous pyoderma gangrenosum as the presenting sign of fatal acute myelogenous leukemia. Leuk Lymphoma. (2006) 47:147–50. doi: 10.1080/10428190500254299, PMID: 16321840

[15] Maglie R Genovese G Solimani F Guglielmo A Pileri A Portelli F. Immune-mediated dermatoses in patients with Haematological malignancies: a comprehensive review. Am J Clin Dermatol. (2020) 21:833–54. doi: 10.1007/s40257-020-00553-9, PMID: 32813229 PMC7679319

[16] Lepelletier C Bouaziz JD Rybojad M Bagot M Georgin-Lavialle S Vignon-Pennamen MD. Neutrophilic dermatoses associated with myeloid malignancies. Am J Clin Dermatol. (2019) 20:325–33. doi: 10.1007/S40257-018-00418-2 30632096

[17] Brooklyn TN Williams AM Dunnill MGS Probert CS. T-cell receptor repertoire in pyoderma gangrenosum: evidence for clonal expansions and trafficking. Br J Dermatol. (2007) 157:960–6. doi: 10.1111/J.1365-2133.2007.08211.X, PMID: 17935516

[18] Kridin K Damiani G Ludwig RJ Tzur Bitan D Cohen AD. Estimating the odds of ulcerative colitis-associated pyoderma Gangrenosum: a population-based case-control study. Dermatology. (2021) 237:323–9. doi: 10.1159/000512931, PMID: 33647909

[19] Maronese CA Pimentel MA Li MM Genovese G Ortega-Loayza AG Marzano AV. Pyoderma Gangrenosum: An updated literature review on established and emerging pharmacological treatments. Am J Clin Dermatol. (2022) 23:615–34. doi: 10.1007/S40257-022-00699-8, PMID: 35606650 PMC9464730

[20] An ZY Fu H He Y Zhu X Huang QS Wu J. Projected global trends in hematological malignancies: incidence, mortality, and disability-adjusted life years from 2020 to 2030. Blood. (2023) 142:3810. doi: 10.1182/BLOOD-2023-187856

[21] Langan SM Groves RW Card TR Gulliford MC. Incidence, mortality, and disease associations of pyoderma gangrenosum in the United Kingdom: a retrospective cohort study. J Invest Dermatol. (2012) 132:2166–70. doi: 10.1038/jid.2012.130, PMID: 22534879

[22] Höfler M. The Bradford Hill considerations on causality: a counterfactual perspective. Emerg Themes Epidemiol. (2005) 2:11. doi: 10.1186/1742-7622-2-11, PMID: 16269083 PMC1291382

[23] Cohen AD Dreiher J Regev-Rosenberg S Yakovson O Lieberman N Goldfracht M. The quality indigators program in Clalit Health Services: the first decade. Harefuah. (2010) 149:204–209, 265. PMID: 20812490

